# Annual acknowledgement of manuscript reviewers

**DOI:** 10.1186/s13104-015-0980-8

**Published:** 2015-02-04

**Authors:** Christopher Foote

**Affiliations:** BioMed Central, Floor 6, 236 Gray’s Inn Road, London, WC1X 8HB UK

## Abstract

The editors of *BMC Research Notes* would like to thank all our reviewers who have contributed to the journal in Volume 7 (2014).

**Jenny Abanto**

Brazil

**Nima Abbassi-Ghadi**

United Kingdom

**Tajeldin Abdallah**

Sudan

**Noriham Abdullah**

Malaysia

**Majedah Abdul-Rasoul**

Kuwait

**Shahab Abid**

Pakistan

**Redouane Abouqal**

Morocco

**Jônatas Abrahão**

Brazil

**Florence Abravanel**

France

**Amit Acharya**

United States of America

**Cheryl Ackert-Bicknell**

United States of America

**George Acquaah-Mensah**

United States of America

**Samuel E. K. Acquah**

Ghana

**Rodney Adam**

United States of America

**Ishag Adam**

Sudan

**Lukasz Adaszek**

Poland

**Adebode Adekanle**

Nigeria

**Miriam Adhikari**

South Africa

**Dan Agranoff**

United Kingdom

**Anurag Agrawal**

India

**Ole Ahlehoff**

Denmark

**Norazah Ahmad**

Malaysia

**Raja Ahmad**

Malaysia

**Suhail Ahmad**

Kuwait

**Tahera Ahmed**

Bangladesh

**Zaghloul Ahmed**

United States of America

**Kamran Mahmood Ahmed Aziz**

Saudi Arabia

**Tom Ainscough**

United Kingdom

**Jane-Francis Akoachere**

Cameroon

**Abdurrahman Aktumsek**

Turkey

**Raid Alany**

United Kingdom

**Salah Al-Awaidy**

Oman

**Steven Albert**

United States of America

**Max Alekseyev**

United States of America

**Atalay Alem**

Ethiopia

**Abebe Alemu**

Ethiopia

**Helene Alexanderson**

Sweden

**Abdullah Alherbish**

Saudi Arabia

**Abdel Aziem Ali**

Sudan

**Mohammad Ali**

India

**Kamran Alimoghaddam**

Iran

**Guillaume Alinier**

Qatar

**Muktar Aliyu**

United States of America

**Omar Alkandari**

Canada

**Nadim Alkharouf**

United States of America

**David Allan**

Canada

**Lindsey Allan**

United Kingdom

**Eugenia Allegra**

Italy

**Paul Allison**

Canada

**Guillermina Almazan**

Canada

**Adelaide Almeida**

Portugal

**Nuno Almeida**

Portugal

**Paulo Almeida**

Portugal

**Jose M Almerich**

Spain

**Luis Alou**

Spain

**Harun Alp**

Turkey

**Jaffar Al-Tawfiq**

Saudi Arabia

**Cosme Alvarado-Esquivel**

Mexico

**Stephen Alway**

United States of America

**Anton Amann**

Austria

**Srikanth Ambati**

United States of America

**Tarek Amin**

Egypt

**Neda Aminzadeh**

Iran

**Oluwatoyin Amira**

Nigeria

**Takasuke Amizuka**

Japan

**Ruopeng An**

United States of America

**Robin Anderson**

United States of America

**Jan Andersson**

Sweden

**Emmanouil Angelakis**

France

**Mariana Angoa-Perez**

United States of America

**Emiliano Antiga**

Italy

**Giulia Antoniali**

Italy

**Filippo Antonini**

Italy

**Barbara Antuna-Puente**

Mexico

**Dimitrios Anyfantakis**

Greece

**Edward Araujo Júnior**

Brazil

**Mehdi Arbabi Ghahroudi**

Canada

**Sophie Arbefeville**

United States of America

**Ana Areia**

Portugal

**Kristina Areskoug Josefsson**

Sweden

**Arianna Aricò**

Italy

**Martin Aringer**

Germany

**Nikolaos Arkadopoulos**

Greece

**Miguel Armengot**

Spain

**Juan Ignacio Arraras**

Spain

**Benedict Oppong Asamoah**

Sweden

**Elad Asher**

Israel

**Shervin Assari**

United States of America

**Stelios Assimakopoulos**

Greece

**Kenneth Aston**

United States of America

**Muhammad Atif**

Pakistan

**Bridget Atkins**

United Kingdom

**Matias Attene Ramos**

United States of America

**Halimah Awang**

Malaysia

**Inge Axelsson**

Sweden

**Makoto Ayabe**

Japan

**Nuno Azevedo**

Portugal

**Alejandro Babayan**

Mexico

**Giridhara R Babu**

India

**Elfriede Marianne Bacchi**

Brazil

**Aranya Bagchi**

United States of America

**Theeshan Bahorun**

Mauritius

**Henry Bailey**

Trinidad and Tobago

**Patricia Bailey**

United States of America

**Simon Bailey**

United Kingdom

**Jillian Bainard**

Canada

**Alan Bainbridge**

United Kingdom

**Ming-Jong Bair**

Taiwan

**Abdelbari Baitar**

Belgium

**Sarita Bajaj**

India

**Tim Baker**

Sweden

**Bita Bakhshi**

Iran

**Nicola Baldini**

Italy

**Piyatissa Laksman Bandara**

Sri Lanka

**Amitav Banerjee**

India

**Anjan Banerjee**

United Kingdom

**Tuhina Banerjee**

India

**Maggi Banning**

United Kingdom

**Vishwas Bapat**

India

**Ashoktaru Barat**

India

**John Barbara**

United Kingdom

**Francois Barbier**

France

**Lesley Barclay**

Australia

**Janine Barden-O'Fallon**

United States of America

**Marinus Barnard**

South Africa

**Chris Barnhart**

United States of America

**Jacques Barrat**

France

**Paul D Bartels**

Denmark

**Tom Bashford**

United Kingdom

**Amal Bashir**

Sudan

**Fernanda Basso**

Brazil

**Jayme Bastos**

Brazil

**Devraj Basu**

United States of America

**Veronique Bataille**

United Kingdom

**Judy Bauer**

Australia

**Richard Bayford**

United Kingdom

**Joel Bazira**

Uganda

**Sara Becker**

United States of America

**Heleen Beckerman**

Netherlands

**Simon Beddows**

United Kingdom

**Ronen Beeri**

Israel

**Prakash Behere**

India

**Ahmad Behroozian**

Iran

**Lynn Bekris**

United States of America

**Jose Belizario**

Brazil

**Ashwin Belle**

United States of America

**Jeremy Bellien**

France

**Jibril Oyekunle Bello**

Nigeria

**Joseph Belter**

United States of America

**Yeshambel Belyhun**

Ethiopia

**Sompop Bencharit**

United States of America

**Marlene Benchimol**

Brazil

**Brian Bennett**

United States of America

**Emmanuel Benoist**

Switzerland

**Charlotte Benson**

United Kingdom

**Richard Bentham**

Australia

**James Berkley**

Kenya

**Karine Bernard**

Canada

**Claire Bertelli**

Switzerland

**Philippe Berthelot**

France

**Getenet Beyene**

Ethiopia

**Gajananda Bhandari**

Nepal

**V Bhanuprakash**

India

**Tej Bhat**

India

**Mrinal Bhave**

Australia

**Bishwajit Bhowmik**

Bangladesh

**Fantahun Biadglegne**

Ethiopia

**Massimiliano Biagini**

Italy

**Paolo Biban**

Italy

**Kyle Bibby**

United States of America

**Mark Bicket**

United States of America

**Annemiek Bielderman**

Netherlands

**Andras Bikov**

Hungary

**Steven Billings**

United States of America

**Stephanie Bingham**

United States of America

**Karen Birch**

United Kingdom

**Hiroko Bissen-Miyajim**

Japan

**Glenn Björklund**

Sweden

**Bronagh Blackwood**

United Kingdom

**Roman Blaheta**

Germany

**Shona Blair**

Australia

**Chellakkan S Blesson**

United States of America

**Jon Block**

United States of America

**Jochen Blom**

Germany

**Richard Bodnar**

United States of America

**Levente Bodrossy**

Austria

**Gregory Bogdanis**

Greece

**Stefan Böhringer**

Netherlands

**Venkatesh Bollina**

Canada

**Martin Bommer**

Germany

**Mario Bonet De La Nuez**

Spain

**Sebastiano Bonventre**

Italy

**Wallapa Boonrod**

Thailand

**Andrew Booth**

United Kingdom

**Anwar Borai**

Saudi Arabia

**Guglielmo Borgia**

Italy

**Victor Borja-Aburto**

Mexico

**Sotirios Botaitis**

Greece

**Rami Bou Khalil**

France

**Marc Boutry**

Belgium

**James Bower**

United States of America

**Josephine Bowles**

Australia

**Lyudmila Boyanova**

Bulgaria

**Cynthia Boyd**

United States of America

**Bruno Leonardo Bozaquel Morais**

Brazil

**Alexander U. Brandt**

Germany

**Anne Lise Brantsaeter**

Norway

**Philip Britton**

Australia

**Claudia Brodskyn**

Brazil

**Evelyn Bromet**

United States of America

**Shobha Broor**

India

**Stig Brorson**

Denmark

**Sarah Brown**

United Kingdom

**Colette Browning**

Australia

**Sjoerd M Bruijn**

Netherlands

**Natale Daniele Brunetti**

Italy

**Michal Brylinski**

United States of America

**Edyta Brzoska**

Poland

**Le Bu**

China

**Thomas Buckley**

United States of America

**Stephanie Budge**

United States of America

**Pierre Buekens**

United States of America

**Ruben Buendia**

Sweden

**Jakob Burcharth**

Denmark

**Emily Burger**

Norway

**Brian Burnes**

United States of America

**Sarah Burr**

Gambia

**Christophe Burucoa**

France

**Sakib Burza**

India

**Florian Busch**

Australia

**Mathias Büttner**

Germany

**Walter Buzina**

Austria

**Jenny Byrne**

United Kingdom

**Jens Byskov**

Denmark

**Shaojiang Cai**

China

**Birsen Cakir**

Turkey

**Emma Callegari**

Australia

**Steven Callens**

Belgium

**Darren Calley**

United States of America

**Raffaele Calogero**

Italy

**Vitaliano Cama**

United States of America

**Maria Jesús Cañal**

Spain

**Rossella Canese**

Italy

**Jose Manuel Cano Arias**

Finland

**Deon Canyon**

United States of America

**Dengfeng Cao**

China

**Maurizia Capuzzo**

Italy

**Luis Caraballo**

Colombia

**Giuseppe Cardillo**

Italy

**Alison Carlisle**

United Kingdom

**Amtul Carmichael**

United Kingdom

**Jan Carney**

United States of America

**Carolyn Anne Carr**

United Kingdom

**Moacir Carretta Júnior**

Brazil

**Paola Caruso**

Italy

**Luis Carvalho**

United States of America

**Beatrice Casini**

Italy

**Leandro Castellano**

United Kingdom

**Maria Jesus Casuso Holgado**

Spain

**Ferrán Catalá-López**

Spain

**Cengiz Cavusoglu**

Turkey

**Eduardo A. Ceccarelli**

Argentina

**S.Burcak Cehreli**

Turkey

**Mevlut Celikoglu**

Turkey

**Nuno Cerca**

Portugal

**Ismail Ceyhan**

Turkey

**Hassen Chaka**

Ethiopia

**Melanie Chalder**

United Kingdom

**Mary Chambers**

United Kingdom

**Ananda Chandrasekara**

Australia

**Chia-Ming Chang**

Taiwan

**Ching-Wei Chang**

United States of America

**Ming-Hong Chang**

Taiwan

**Tzu-Hao Chang**

Taiwan

**Yoon Soo Chang**

South Korea

**Neil Chapman**

United Kingdom

**Fadi Charchar**

Australia

**Gregory Chasson**

United States of America

**Vijay Chava**

India

**Joseph Cheatwood**

United States of America

**Geng Chen**

China

**Meng-Hua Chen**

China

**Elizabeth Chen**

United States of America

**Xiangyan Chen**

Hong Kong

**Ying-Chieh Chen**

Taiwan

**Linyi Chen**

Taiwan

**Wenan Chen**

United States of America

**Benoît Chénais**

France

**Louis Cheung**

Hong Kong

**Mike W.-L. Cheung**

Singapore

**Tzen-Yuh Chiang**

Taiwan

**Chandeshwari Chilampalli**

United States of America

**Jennifer Chipps**

South Africa

**King-Wah Chiu**

Taiwan

**Haruhiko Cho**

Japan

**Bowornsilp Chowchuen**

Thailand

**Elisabeth Chroni**

Greece

**Shih-Chang Chuang**

Taiwan

**Secundino Cigarran**

Spain

**Irina Cleemput**

Belgium

**John Cleland**

United Kingdom

**Peter Clifton**

Australia

**Jared Coburn**

United States of America

**Chris Coggins**

United States of America

**Kim Connelly**

Canada

**Nicholas Connors**

United States of America

**Jean-Michel Constantin**

France

**Carla Contaldi**

Italy

**Daniel Cook**

United States of America

**David Cooke**

United Kingdom

**Mary Coolbaugh-Murphy**

United States of America

**Jason Corburn**

United States of America

**Ricardo Correa**

United States of America

**David Cosgrove**

United Kingdom

**Céire Costelloe**

United Kingdom

**Joanne Coster**

United Kingdom

**Vasile Cozma**

Romania

**Holger Cramer**

Germany

**Andrew Croftys**

Japan

**Ray Croucher**

United Kingdom

**Fulvio Cruciani**

Italy

**Rik Crutzen**

Netherlands

**Yendelela Cuffee**

United States of America

**Juan Cui**

United States of America

**Xinping Cui**

United States of America

**Bibiana Cujec**

Canada

**Antonio Curra**

Italy

**Douglas Curran-Everett**

United States of America

**Clare Cutland**

South Africa

**Hannah Dada-Adegbola**

Nigeria

**Akram Da'Darah**

United States of America

**Elizabeth Daher**

Brazil

**Jesper Dahlgaard**

Denmark

**Jeanette Daly**

United States of America

**Till Dammaschke**

Germany

**M. Carolina Danovaro-Holliday**

United States of America

**Shrinivas Darak**

India

**Srijit Das**

Malaysia

**Sulochana Das**

India

**Abdullah Dasgic**

Turkey

**Giuseppe Dattilo**

Italy

**Seema Daud**

Pakistan

**John Davey**

United Kingdom

**William Davies**

United Kingdom

**Gill Davies**

United Kingdom

**Renée de Cremoux**

France

**Fusina De Graaff**

Netherlands

**Astrid de Greeff**

Netherlands

**Birgitta de Jong**

Sweden

**Blandine de Lauzon-Guillain**

France

**Claudio Andre Barbosa de Lira**

Brazil

**Nicolò de Manzini**

Italy

**Giuseppe Vittorio De Socio**

Italy

**Jan De Waele**

Belgium

**Anna Dean**

Switzerland

**Elizabeth Dean**

Canada

**Chitrita Debroy**

United States of America

**Martin Dedicoat**

United Kingdom

**Wanchai De-Eknamkul**

Thailand

**Savas Deftereos**

Greece

**Aldo Dekker**

Netherlands

**Manuel Delgado**

Spain

**Hélène Delisle**

Canada

**Stefaan Demarest**

Belgium

**Ahmet Demirok**

Turkey

**Meaza Demissie**

Ethiopia

**Ning Deng**

China

**Tom Dening**

United Kingdom

**Kebede Deribe**

Ethiopia

**Hay Derkx**

Netherlands

**Yadeta Dessie**

Ethiopia

**Ferdinando Di Cunto**

Italy

**Pietro Di Lena**

Italy

**Fabiano Di Marco**

Italy

**Patrick Di Martino**

France

**Paolo Di Tommaso**

Spain

**Ricardo Dias**

Brazil

**James Dickinson**

Canada

**Eric Dieperink**

United States of America

**Michele Diniz**

Brazil

**Ener Cagri Dinleyici**

Turkey

**Konstantinos Diplaris**

Greece

**Joerg Dirmaier**

Germany

**Betty Dodet**

France

**Maman Dogba**

Canada

**Ibrahim Domian**

United States of America

**Davide Maria Donati**

Italy

**Ravi Kiran Donthu**

United States of America

**Karel Dorey**

United Kingdom

**Marcus Dörr**

Germany

**David Dosoo**

Ghana

**Julia Downing**

Uganda

**Paul Drain**

United States of America

**Alex Dregan**

United Kingdom

**Ton Drenthen**

Netherlands

**Gregory Dressler**

United States of America

**Ian Driver**

United Kingdom

**Zhong-Hui Duan**

United States of America

**Deep Dudeja**

United States of America

**Mark Duffett**

Canada

**Vanja Dukic**

United States of America

**Andrew Duncan**

United States of America

**Pavel Dundr**

Czech Republic

**Eileen Dunne**

Australia

**Xavier Duralde**

United States of America

**Jeffery Dusek**

United States of America

**Shanta Dutta**

India

**Melanie Duval**

United States of America

**Jon Duvick**

United States of America

**Chandradhar Dwivedi**

United States of America

**Barney Dwyer**

United States of America

**Deborah Eastwood**

United Kingdom

**Jose Echevarria**

Spain

**Thorsten H. Ecke**

Germany

**Michael Eddleston**

United Kingdom

**Tewodros Eguale**

Canada

**William Ehlers**

United States of America

**Lars Eisen**

United States of America

**Alvin Eisner**

United States of America

**Husamettin Ekici**

Turkey

**Martins Ekor**

Ghana

**Mona El Lawindi**

Egypt

**Ahmed El Nahas**

Egypt

**Azza El Nouman**

Egypt

**Theodoros Eleftheriadis**

Greece

**Elhassan Elhassan**

Sudan

**Ron Eliashar**

Israel

**Jeffrey Elmendorf**

United States of America

**Gülsüm Özlem Elpek**

Turkey

**Maged El-Setouhy**

Saudi Arabia

**Solomon Emishaw**

Ethiopia

**Mengistu Endris**

Ethiopia

**Charis Eng**

United States of America

**Alexandru Eniu**

Romania

**Sabrina Epiphanio**

Brazil

**Carlos Luis Errando**

Spain

**Shankar Esaki Muthu**

Malaysia

**Antonio Escobar**

Spain

**Neda Esmailzadehha**

Iran

**Ciro Estrada-Chavez**

Mexico

**Ben Evans**

Canada

**Angelica Facoetti**

Italy

**Cyntia Fadel-Picheth**

Brazil

**Vicenç Falcó**

Spain

**Marco Falda**

Italy

**Francisco Fanjul**

Spain

**Daniel Farias**

Brazil

**Antonio Farina**

Italy

**Guido Favia**

Italy

**Lambert Felix**

United Kingdom

**Quan Zhou Feng**

China

**Yifan Feng**

China

**Yaoyu Feng**

China

**Mybera Ferizi**

Serbia

**Pedro Fernandes**

Portugal

**Lucie Fernandez**

France

**Xavier Fernández-Aguilar**

Spain

**Jose Francisco Fernández-Garayzábal**

Spain

**M. Rohan Fernando**

United States of America

**Alisdair Fernie**

Germany

**Nelia Ferraria**

Portugal

**Ubirajara Ferreira**

Brazil

**Fernando Ferrero**

Argentina

**Leah Ferrucci**

United States of America

**Taciane Finatto**

Brazil

**Rick Finch**

United States of America

**Alessandro Finkelsztejn**

Brazil

**Felix Fischer**

Germany

**Aryeh Fischer**

United States of America

**Giulia Fiscon**

Italy

**Mary Flatley**

United Kingdom

**Yannick Fleury**

France

**Lars Folkestad**

Denmark

**Virginia Fonner**

United States of America

**Cuisle Forde**

Ireland

**Ola Forslund**

Sweden

**Roberto Foschino**

Italy

**Russell Foxall**

Portugal

**Sylvie Fraitag**

France

**M. Pilar Francino**

Spain

**Richard Francis**

Australia

**Arthur Frank**

United States of America

**Samuel Frank**

United States of America

**Jan Erik Frantsvåg**

Norway

**Willard Freeman**

United States of America

**Michael French**

United Kingdom

**Erwin Frey**

Germany

**Niels Frimodt-Moller**

Denmark

**Marvin Fritzler**

Canada

**Junfen Fu**

China

**Yong Fu**

United States of America

**Ezequiel Fuentes-Panana**

Mexico

**Takamitsu Fujimaki**

Japan

**Ricardo Fujiwara**

Brazil

**Yasuo Fukano**

Japan

**Seiji Fukumoto**

Japan

**Hidetada Fukushima**

Japan

**Franco Fulciniti**

Switzerland

**Andre Fuly**

Brazil

**Shahinaz Gadalla**

United States of America

**Mohammed Gadour**

Sudan

**Jan Gaertner**

Germany

**Gianluca Gaidano**

Italy

**Thomas Gaj**

United States of America

**Umberto Galderisi**

Italy

**Phillip Gallo**

United States of America

**Moses Galukande**

Uganda

**George Galyfos**

Greece

**Maria Gamaletsou**

Greece

**Michael Gantier**

Australia

**You-Shui Gao**

China

**Mingming Gao**

United States of America

**Dana Garcia**

United States of America

**Ana L. Garcia-Perez**

Spain

**Shea Gardner**

United States of America

**Scott Garman**

United States of America

**Russell Garwood**

United Kingdom

**Gasim Gasim**

Sudan

**M Carolyn Gates**

United Kingdom

**Trent Gause**

United States of America

**Yngvar Gauslaa**

Norway

**Lidia Gavic**

Croatia

**Ezra Gayawan**

Nigeria

**Philippe Gayral**

France

**Ejigu Gebeye**

Ethiopia

**Endrias Gebremedhin**

Ethiopia

**Amit Gefen**

Israel

**Georgios Kouklakis**

Greece

**Eveline Geubbels**

Tanzania

**Akhgar Ghassabian**

United States of America

**Ravindra Ghooi**

India

**Rohini Ghosh**

India

**Giannis Giakas**

Greece

**Christoforos Giannaki**

Cyprus

**Phil Giffard**

Australia

**Elena Giglia**

Italy

**Hazel Gilbert**

United Kingdom

**Sophie Gillain**

Belgium

**Dominique Giorgi**

France

**Bruno Giraudeau**

France

**Fiseha Girma**

Ethiopia

**Konstantinos Gkagkalidis**

Greece

**Melissa Gladstone**

United Kingdom

**Stefan Gluck**

United States of America

**Apul Goel**

India

**Karen Goldstein**

United States of America

**Srinivas Goli**

India

**Mário Gomes**

Portugal

**Pedro Gómez**

Spain

**Vivian Gonzalez**

United States of America

**Daniel Gonzalez Mendoza**

Mexico

**Manuel González-Sánchez**

Spain

**Fidel González-Torralva**

Spain

**Dawn Goodwin**

United Kingdom

**Stephen Goodwin**

United States of America

**Vijayaprasad Gopichandran**

India

**Alpa Gor**

India

**Ariela Gordon-Shaag**

Israel

**Marga Goris**

Netherlands

**Rashmi Goswami**

Canada

**Tomoko Goto**

Japan

**Riccardo Gottardi**

United States of America

**Evanthia Gouveri**

Greece

**Emmanuelle Graciet**

Ireland

**Pippa Graham**

United Kingdom

**Stephen Grant**

United Kingdom

**Stephen Graves**

Australia

**Mike Gray**

United Kingdom

**William Gray**

United Kingdom

**Esther Green**

Canada

**Keri Griffin**

United States of America

**Nikolaos Grigoriadis**

Greece

**Tamasine Grimes**

Ireland

**Adriaan O Grobbelaar**

United Kingdom

**Dina Grohmann**

Germany

**Richard Grove**

United Kingdom

**Alexandra Gruss**

France

**Tore Gude**

Norway

**Joao Guerreiro**

Brazil

**Jian-Fang Gui**

China

**Daniel Guillaume**

United States of America

**Alexandre Guimarães**

Brazil

**Josee Guindon**

United States of America

**Oriol Guitart Pla**

Spain

**Deb Gumucio**

United States of America

**Sumanth Gunupati**

India

**Wen-Wu Guo**

China

**Robert Hadden**

United Kingdom

**Dimitra Hadjipavlou**

Greece

**Walter Haefeli**

Germany

**Bruno Hagenbuch**

United States of America

**Ghulam Haider**

Pakistan

**Saida Haider**

Pakistan

**Lukman Hakim**

Belgium

**Marta Hallak**

Argentina

**Oskar Hallatschek**

United States of America

**Katherine Hammer**

Australia

**Klaus Hamprecht**

Germany

**Rasha Hamza**

Egypt

**Yuyan Han**

United States of America

**Xiang-Yang Han**

United States of America

**Rima Hanna Wakim**

Lebanon

**Thomas Hänscheid**

Portugal

**Robert Hansebout**

Canada

**Kristina Hanspers**

United States of America

**Feras Hantash**

United States of America

**Philippe Hantson**

Belgium

**Akira Hara**

Japan

**Edna Hardeman**

Australia

**Stephen Hardies**

United States of America

**Alexandre Harlé**

France

**Diane Harper**

United States of America

**Ann Harris**

United States of America

**James Harrison**

United States of America

**Dominik Hartl**

Germany

**John Hartung**

United States of America

**Zahra Hasan**

Pakistan

**Vincent Hascall**

United States of America

**Ziad Hassan**

Hungary

**Takashi Hatano**

Japan

**Bisong Haupt**

United States of America

**Radim Havelek**

Czech Republic

**Dave Hayes**

Canada

**Nicholas Hellenthal**

United States of America

**Helena Helmby**

United Kingdom

**Julie Henderson**

Australia

**Moon Her**

South Korea

**Klemens Hertel**

United States of America

**Des Higgins**

Ireland

**Taichi Hikichi**

Japan

**Ralf Axel Hilger**

Germany

**Srinivasa Hiresave**

India

**Gökben Hizli Sayar**

Turkey

**Tiny Motlatso Hlokwe**

South Africa

**Shinn-Ying Ho**

Taiwan

**Thierry Hoareau**

South Africa

**Jan Hoek**

United States of America

**Are Holen**

Norway

**Jennifer Hollowell**

United Kingdom

**Johannes Holmen**

Norway

**Marja Holmila**

Finland

**Rodney Honeycutt**

United States of America

**Peiying Hong**

Saudi Arabia

**Norio Horie**

Japan

**Hidehito Horinouchi**

Japan

**Thomas Hörnschemeyer**

Germany

**Xueqing Hsu**

China

**Chia-Lang Hsu**

Taiwan

**Huey-Miin Hsueh**

Taiwan

**Dongsheng Hu**

China

**Kyumg-Seok Hu**

South Korea

**Xin Huang**

United States of America

**Lei Huang**

United States of America

**Shu-Hong Huang**

China

**Zhuoyi Huang**

United States of America

**Cedric Hubeau**

United States of America

**Roman Huber**

Germany

**Jan Hubert**

Czech Republic

**Patricia Hudelson**

Switzerland

**Will Hueston**

United States of America

**Christopher Hughes**

United States of America

**Winston Huh**

United States of America

**David Humphreys**

Australia

**Asghar Hussain**

Pakistan

**Gisela Hussey**

United States of America

**Brent Hutto**

United States of America

**Arnaud Huvet**

France

**Sylvia Hysong**

United States of America

**Roberta Iatta**

Italy

**Philip Ibinaiye**

Nigeria

**Jamaiah Ibrahim**

Malaysia

**Hideaki Ijichi**

Japan

**Hisao Imai**

Japan

**Mohammad Zafar Imam**

Bangladesh

**Michiaki Imamura**

United States of America

**Shinsaku Imashuku**

Japan

**Aamer Imdad**

United States of America

**Jose Imparato**

Brazil

**Toru Inami**

Japan

**Julia Indik**

United States of America

**Annette Ingeman**

Denmark

**Maia Ingram**

United States of America

**Amy Inselman**

United States of America

**Cherdsak Iramaneerat**

Thailand

**Atsushi Irisawa**

Japan

**Marcello Iriti**

Italy

**Michael Iroezindu**

Nigeria

**James Ironside**

United Kingdom

**Yasuko Ishida**

United States of America

**Masakazu Ishii**

Japan

**Nabil Ismaili**

Morocco

**Takeshi Isobe**

Japan

**Yasuhiro Ito**

Japan

**Jaroslaw Jacak**

Austria

**Felice Jacka**

Australia

**Pamela Jackson**

United States of America

**Daral Jackwood**

United States of America

**David Jacobs**

United States of America

**Jan Jacobs**

Belgium

**George Jacoby**

United States of America

**Mario Jacques**

Canada

**Shalini Jadia**

India

**Lamin E.S. Jaiteh**

Gambia

**Bawo James**

Nigeria

**Nicol Janecko**

Canada

**Robert Janulczyk**

Italy

**Nicolas Janus**

France

**Penny Jeggo**

United Kingdom

**Pratap Jena**

India

**Andreas Jenke**

Germany

**Sue Jenkins**

Australia

**Viala Jérôme**

France

**James Jester**

United States of America

**Vishal Jhanji**

India

**Yu Jiang**

United States of America

**Jie Jin**

China

**Yaping Jin**

China

**Andrei Joaquim**

Brazil

**Ane Johannessen**

Norway

**Andrew John**

United States of America

**Simone Johner**

Germany

**Graham Johnson**

Australia

**Kay Jones**

Australia

**Philippe Jorens**

Belgium

**Noyal Joseph**

India

**Carlos Juan Nicolau**

Spain

**Sebastian Jud**

Germany

**John Jue**

Canada

**Gyungah Jun**

United States of America

**Sini Junttila**

Finland

**Susanne Kaae**

Denmark

**Billingsley Kaambwa**

Australia

**Sushi Kadanakuppe**

India

**Jun-Ichi Kadokawa**

Japan

**Ignatius Kakande**

Rwanda

**László Kalabay**

Hungary

**Adina Kalet**

United States of America

**Dorota Kaleta**

Poland

**Natarajaseenivasan Kalimuthusamy**

India

**Nikolaos Kalinderis**

Greece

**Antonia Kaltsatou**

Greece

**Joshua Kamani**

Nigeria

**Kenya Kamimura**

Japan

**Eli Kaminuma**

Japan

**Shigeru Kamiya**

Japan

**Sandeep Kumar Kanwal**

India

**Murat Kapan**

Turkey

**Nathan Kapata**

Zambia

**Sunil Karande**

India

**Cumali Karatoprak**

Turkey

**Elina Kari**

United States of America

**Samuel Kariuki**

Kenya

**Rajendra Karkee**

Nepal

**Stephen Karl**

United States of America

**Linnea Karlsson**

Finland

**Thomas Karn**

Germany

**Nikolaos Karpathakis**

Greece

**Saiful Anuar Karsani**

Malaysia

**Sathya Karunananthan**

Canada

**Panduka Karunanayake**

Sri Lanka

**David Kateete**

Uganda

**Jun Kato**

Japan

**Pankaj Kaul**

United Kingdom

**Method Kazaura**

Tanzania

**Hannah Keage**

Australia

**Scott Kellerman**

United States of America

**Roger Kelley**

United States of America

**Angela Kelly-Hanku**

Papua New Guinea

**Troy Kemp**

United States of America

**Kristina Kendall**

United States of America

**Laurence Kennedy**

United States of America

**Lindsey Kennedy**

United States of America

**Andrew Kerkhoff**

United States of America

**Ryan Kerney**

United States of America

**Eero Kerosuo**

Norway

**Kassahun Ketema**

Ethiopia

**Daniel Keyler**

United States of America

**Anfumbom Kfutwah**

Cameroon

**Asaduzzaman Khan**

China

**Nancy Khardori**

United States of America

**Mustafa Khasraw**

Australia

**Adam Kiefer**

United States of America

**Elesban Kihuba**

Kenya

**Cenk Kilic**

Turkey

**Bong-Soo Kim**

South Korea

**Hyung Joong Kim**

South Korea

**Gi Jin Kim**

South Korea

**Ku Sang Kim**

South Korea

**Nayoung Kim**

South Korea

**Seung-Ki Kim**

South Korea

**Tae Hun Kim**

South Korea

**Tae-Min Kim**

South Korea

**Helen Kimbi**

Cameroon

**Leigh Kinsman**

Australia

**Inge Kirchberger**

Germany

**Hiroyuki Kirikoshi**

Japan

**Stefan Kirov**

United States of America

**Marilynn Kish-Molina**

United States of America

**Masahiro Kitada**

Japan

**Mary Ellen Kleinhenz**

United States of America

**Alexa Klettner**

Germany

**Gert Klug**

Austria

**Caprice Knapp**

United States of America

**Joseph Knapper**

United States of America

**Ketevan Kobaidze**

United States of America

**Ichizo Kobayashi**

Japan

**Naomi Kobayashi**

Japan

**Nobumichi Kobayashi**

Japan

**Kord Kober**

United States of America

**Jean-Pierre Kocher**

United States of America

**Angela Koech**

Kenya

**Ruchika Kohli**

Kenya

**Tsuyoshi Koide**

Japan

**Hitoshi Komatsuzawa**

Japan

**Lei Kong**

China

**Justin Konje**

United Kingdom

**George Konstantinou**

United States of America

**Mirjam Kooistra-Smid**

Netherlands

**Nicholas George Kounis**

Greece

**Argyro Krase**

Greece

**Anand Krishnan**

India

**Lasse Sommer Kristensen**

Denmark

**Shosei Kubota**

Japan

**Iwao Kukimoto**

Japan

**Shalini Kulasingam**

United States of America

**Ambuj Kumar**

United States of America

**Mahendra Kumar**

United States of America

**Sanjay Kumar**

India

**Shyamesh Kumar**

United States of America

**Shalini Kumar**

United States of America

**Yashwant Kumar**

India

**Mohamed Kurer**

United Kingdom

**Toshihide Kuroki**

Japan

**Mohankumar Kurukumbi**

United States of America

**Koichi Kusuhara**

Japan

**Marie-Aleth Lacaille-Dubois**

France

**Harrie Lafeber**

Netherlands

**Chih-Cheng Lai**

Taiwan

**Hsin-Chih Lai**

Taiwan

**Ching-Huang Lai**

Taiwan

**Wazir Lakra**

India

**Umashankar Lakshmanadoss**

United States of America

**Luke Simon Lambeth**

Australia

**Antonietta Lambiase**

Italy

**Danette Langbecker**

Australia

**Rupert Langer**

Switzerland

**Tanja Langsenlehner**

Austria

**Matthew Lanktree**

Canada

**Terence Lao**

Hong Kong

**Pamela Larsen**

United States of America

**Anders Larsson**

Sweden

**Rauf Latif**

United States of America

**Renat Latipov**

Uzbekistan

**Wei Lau**

China

**Caroline Laurence**

Australia

**Massimiliano Lauria**

Italy

**Lisbeth Laursen**

Denmark

**Eun Jin Lee**

South Korea

**Hsin-Chen Lee**

Taiwan

**King-Teh Lee**

Taiwan

**Eun Bong Lee**

South Korea

**Seon-Woo Lee**

South Korea

**Seonghee Lee**

United States of America

**Rosa LeGood**

United Kingdom

**Loïc Legrand**

France

**Michael Lehker**

United States of America

**Jerome Leis**

Canada

**Louise Lemieux-Charles**

Canada

**Martha Lemnge**

Tanzania

**Aymeric Leray**

France

**Tinne Lernout**

Belgium

**Mathilde Lescat**

France

**Lenard Lesser**

United States of America

**Marilyn Levi**

United States of America

**Phillip Levine**

United States of America

**Ofer Levy**

United Kingdom

**Lene Levy-Storms**

United States of America

**Junling Li**

China

**Gang Li**

United States of America

**Guojun Li**

United States of America

**Jieyue Li**

United States of America

**Fang Li**

United States of America

**Xin-Xu Li**

China

**Mai Suan Li**

Poland

**Ting Li**

United States of America

**Weijiang Li**

China

**Xiujun Li**

China

**Thawornchai Limjindaporn**

Thailand

**Chia-Chi Lin**

Taiwan

**Tung-Liang Lin**

Taiwan

**Song Lin**

China

**Shih-Shun Lin**

Taiwan

**Ming-Wei Lin**

Taiwan

**Adrian Linacre**

Australia

**Ulrich Lindemann**

Germany

**Lars Lindner**

Germany

**Diane Lindsay**

United Kingdom

**Fiona Chun Man Ling**

Ireland

**Gabor Egon Linthorst**

Netherlands

**Kevin Little**

United Kingdom

**Bao Liu**

China

**Pengfei Liu**

China

**Chia-Chyi Liu**

Taiwan

**Jeh-Ping Liu**

United States of America

**Jun Liu**

China

**Kangding Liu**

China

**Ming-Tsan Liu**

Taiwan

**Zheng Liu**

United States of America

**Wei-Cheng Lo**

Taiwan

**Angela Lombardi**

Italy

**Ambrogio Pietro Londero**

Italy

**Thomas London**

United States of America

**William Longabaugh**

United States of America

**Yonggen Lou**

China

**Bruno Louis**

France

**Ray Lovett**

Australia

**Dianne Lowe**

Australia

**Aneta Luczkiewicz**

Poland

**Mario Luini**

Italy

**Andrei Lupas**

Germany

**Winnie Luseno**

United States of America

**Livio Luzi**

Italy

**Xin Ma**

United States of America

**Shuangge Ma**

United States of America

**Duncan Macfarlane**

Hong Kong

**Shingai Machingaidze**

South Africa

**David Macinga**

United States of America

**Sarah Maddocks**

United Kingdom

**Yoshiaki Maeda**

Japan

**Pietro Maffei**

Italy

**Ferdinando Maggioni**

Italy

**Paolo Magistri**

Italy

**Konstantina Magklara**

Greece

**Ganga Mahat**

United States of America

**Duhita Mahatmya**

United States of America

**Mohamed Mahfouz**

Saudi Arabia

**Luciana Furlaneto Maia**

Brazil

**Luciano Carlos Da Maia**

Brazil

**Juraj Majtan**

Slovakia

**Gathsaurie Malavige**

Sri Lanka

**Rahul Malhotra**

Singapore

**Elfatih Malik**

Sudan

**Massimo Mallardo**

Italy

**Romina Mancinelli**

Italy

**Emiri Mandeville**

United States of America

**Paul Manger**

South Africa

**Irena Maniecka-Bryla**

Poland

**Jennifer Manilay**

United States of America

**Mauro Maniscalco**

Italy

**Vangelis Manolopoulos**

Greece

**Ahmed Mansouri**

Germany

**Nitin Mantri**

Australia

**Yaakov Maor**

Israel

**Isabella Marchesi**

Italy

**Federica Marcuzzi**

Italy

**Marketa Mareckova**

Czech Republic

**Andrei Mariandyshev**

Russian Federation

**Michel Marina**

Spain

**Tim Marks**

United Kingdom

**Christine Marosi**

Austria

**Pier Luigi Martelli**

Italy

**John Martens**

Netherlands

**Andrew Martin**

Australia

**Shawn Martin**

United States of America

**Allyson Martinez**

United States of America

**Margarita Martinez-Medina**

Spain

**Carlos Henrique Martins**

Brazil

**Elizabeth Marum**

United States of America

**Kazuto Masamoto**

Japan

**Linnet Masese**

United States of America

**Christopher Mason**

United States of America

**Ghassan Matar**

United States of America

**Karen Mate**

Australia

**Dorthe Matenia**

Germany

**Eugene Mathes**

United States of America

**Joseph Matovu**

Uganda

**Yutaka Matsuoka**

Japan

**Robin Matsuyama**

United States of America

**Paul Matthews**

United States of America

**Barbara Magdalena Matthys**

Switzerland

**Calum Mattocks**

United Kingdom

**Raimonda Matulionyte**

Lithuania

**Marzorati Mauro**

Italy

**Luciano Mavilia**

Italy

**Brian Mavis**

United States of America

**Andreas Mavrogenis**

Greece

**Bernd Mayer**

Austria

**Gherardo Mazziotti**

Italy

**Leonard Mboera**

Tanzania

**Elizabeth McClure**

United States of America

**Scott McComb**

Switzerland

**Pamela McCombe**

Australia

**Kelly McDaniel**

United States of America

**Sheena McGowan**

Australia

**Cecilia McGregor**

United States of America

**Gerald McKinley**

Canada

**Geoff McLachlan**

Australia

**Matthew McMillin**

United States of America

**Peter McMinn**

Australia

**Dennis McNevin**

Australia

**Robert Mecham**

United States of America

**Christopher Medberry**

United States of America

**María Elena Medina-Mora**

Mexico

**Parvin Mehdipour**

Iran

**Judith Meijers**

Netherlands

**Rojelio Mejia**

United States of America

**Anne Mejia-Downs**

United States of America

**Desalew Mekonnen**

Ethiopia

**Roberto Melano**

Canada

**Jacopo Meldolesi**

Italy

**Marcia Mellado Lagarde**

United States of America

**Martin Mempel**

Germany

**Francesca Menconi**

Italy

**Alfonso Mendez-Tenorio**

Mexico

**Getachew Mengistu**

Ethiopia

**Kojo Mensa-Wilmot**

United States of America

**Rainer Merkl**

Germany

**Anthony Metcalf**

United States of America

**Giulio Metro**

Italy

**Marjo Metsäranta**

Finland

**Brian Metscher**

Austria

**Christoph Meyer**

Germany

**Gabriele Meyer**

Germany

**David Meyerholz**

United States of America

**Tammy Meyers**

South Africa

**Ioannis Michos**

Greece

**Piotr Mieczkowski**

United States of America

**Federico Migliore**

Italy

**Massimo Milan**

Italy

**Allison Miller**

United States of America

**Larry Miller**

United States of America

**Andy Miller**

United States of America

**Lillian Min**

United States of America

**Hengameh Mirsepasi**

Denmark

**Avinash Mishra**

India

**KP Mishra**

India

**Mariya Miteva**

United States of America

**Takahiro Mitsui**

Japan

**Aruna Mittal**

India

**Toru Mizuguchi**

Japan

**Abdallah Mkopi**

Tanzania

**Ana Olga Mocumbi**

Mozambique

**Feleke Moges**

Ethiopia

**Fahim Mohamed**

Australia

**Nor Azlin Mohamed Ismail**

Malaysia

**Amir Houshang Mohammad Alizadeh**

Iran

**Abolfazl Mohammadbeigi**

Iran

**Ghorbanali Mohammadi**

Iran

**Gloria María Molina-Salinas**

Mexico

**Christodoulos Monastiriotis**

Greece

**Maurizio Mongia**

Italy

**Akira Monji**

Japan

**Mario Monteiro**

Brazil

**Jesus Montero-Marin**

Spain

**Jonathan Montomoli**

Denmark

**Paolo Montuschi**

Italy

**Arshnee Moodley**

Denmark

**Jacen Moore**

United States of America

**Maria Morgan**

United Kingdom

**Phil Morgan**

United States of America

**Laura Moro**

Spain

**Mustafa Morsy**

United States of America

**Andrea Mosher**

Canada

**Maria Mota**

Portugal

**Panagiotis Moulos**

Greece

**Caroline Moyret-Lalle**

France

**Stephen Mshana**

Tanzania

**Kanigula Mubagwa**

Belgium

**Thomas Muehlbauer**

Germany

**Claudius Mueller**

United States of America

**Gerald Muench**

Australia

**Zulf Mughal**

United Kingdom

**Dieudonne Muhoza**

Rwanda

**Andrew Mujugira**

Uganda

**Vincent Mukkada**

United States of America

**Diriba Muleta**

Ethiopia

**Andargachew Mulu**

Ethiopia

**Silvia Leonor Mundo**

Argentina

**Maria Munne**

Argentina

**Punnagai Munusami**

India

**Chris Murgatroyd**

United Kingdom

**Rita Musetti**

Italy

**Karthikeyan Muthusamy**

India

**Celso Nabais**

Portugal

**Hakan Nacak**

Netherlands

**Simon Nadel**

United Kingdom

**Atsushi J. Nagano**

Japan

**Ramesh Nagarajappa**

India

**Yoji Nagashima**

Japan

**Girish Nair**

Canada

**Rahul Naithani**

India

**Kogenta Nakamura**

Japan

**Katsumasa Nakamura**

Japan

**Shuji Nakano**

Japan

**George Nakos**

Greece

**Luigi Naldi**

Italy

**Rajaie Namas**

United States of America

**Silvia Natoli**

Italy

**Gerardo Nava**

United States of America

**Ana Navarro**

Spain

**Tomas Navarro-Rodriguez**

Brazil

**Fina Navi**

Iran

**Constance Nebendahl**

Germany

**Pradeep Negi**

India

**Praveen Nekkar Rao**

Canada

**Kyle Nelson**

United States of America

**Gopalakrishnan Netuveli**

United Kingdom

**Ines Neves**

Portugal

**Irene Newsham**

United States of America

**Marie Ng**

United States of America

**Ka-Lok Ng**

Taiwan

**Lock Hock Ngu**

Malaysia

**Gordon Nichols**

United Kingdom

**Pin Nie**

China

**Albert Nienhaus**

Germany

**Julie Nightingale**

United Kingdom

**Dimitrios Nikolopoulos**

Greece

**Myra Nimmo**

United Kingdom

**Daisuke Nishi**

Japan

**Yuichiro Nishida**

Japan

**Nifang Niu**

United States of America

**Damir Nizamutdinov**

United States of America

**Carl Erik Nord**

Sweden

**Robert Norgren**

United States of America

**Paul Norman**

United Kingdom

**Davide Normanno**

France

**Emma Norris**

United Kingdom

**Andrew Nosal**

United States of America

**Jan Novy**

Switzerland

**Ali Nuhu**

Nigeria

**Roberto Nuño-Solinís**

Spain

**Uchenna Nwagha**

Nigeria

**Angelo Nyamtema**

Tanzania

**Samuel Robert Nyman**

United Kingdom

**Gerald Obermair**

Austria

**Isidore Obot**

Nigeria

**Beverley O'Brien**

Canada

**John O'Bryan**

United States of America

**Pelin Öçal**

Turkey

**Max O'Donnell**

United States of America

**Aoife O'Donovan**

United States of America

**Ernest Oertli**

United States of America

**Clodagh O'Gorman**

Ireland

**Isiaka Ajani Ogunwande**

Nigeria

**Kazunori Oishi**

Japan

**Dike Ojji**

Nigeria

**Jerry Okal**

Kenya

**Obiekwe Okoye**

Nigeria

**Ana Oliveira**

Portugal

**Robert Olson**

Canada

**Dennis O'Malley**

United States of America

**Torbjorn Omland**

Norway

**Nattawat Onlamoon**

Thailand

**Paolo Onori**

Italy

**Maaike Oosterveer**

Netherlands

**Sjoukje Oosting**

Netherlands

**Francisco Ortega**

Spain

**Angel Ortiz-Pelaez**

United Kingdom

**Pablo L Ortiz-Romero**

Spain

**Lotti Orwelius**

Sweden

**Masahiro Osakabe**

Japan

**Babatunde Osinaike**

Nigeria

**Jan Ostermann**

United States of America

**Nao Otsuka**

Japan

**Junko Otsuki**

Japan

**Tamara Otto**

United States of America

**Constantine Oulis**

Greece

**William Kwame Boakye Ansah Owiredu**

Ghana

**Foluso Owotade**

Nigeria

**Fatih Ozsolak**

United States of America

**Ceyhun Ozyurt**

Turkey

**Juliano Paccez**

South Africa

**Balakrishnan Pachamuthu**

India

**Nicola Page**

South Africa

**Fabio Pagni**

Italy

**Karkala Sreedhara Ranganath Pai**

India

**Eric Pailhoux**

France

**Nirianne Palacpac**

Japan

**Prasit Palittapongarnpim**

Thailand

**Miranda Pallan**

United Kingdom

**Marta Palmieri**

Italy

**Nicola Palmieri**

Austria

**Stefano Palmucci**

Italy

**Juha Paloneva**

Finland

**William Pan**

United States of America

**Katherine Panageas**

United States of America

**Vasileios Panagopoulos**

Greece

**Jagroop Pandhal**

United Kingdom

**Tiziana Pandolfini**

Italy

**Gurusher Panjrath**

United States of America

**Vassiliki Papaevangelou**

Greece

**Masismo Papale**

Italy

**Nikolaos Papanas**

Greece

**Athanasios Papatsoris**

United Kingdom

**Athanasia Papazafiropoulou**

Greece

**Paolo Paredi**

United Kingdom

**Swarup Parida**

India

**Tae Jung Park**

South Korea

**Christopher Parry**

United Kingdom

**Renato Pasquali**

Italy

**Desiderio Passali**

Italy

**Maria Bruna Pasticci**

Italy

**Jennifer Pastorini**

Sri Lanka

**Hemal Patel**

United States of America

**Minal Patel**

United States of America

**Ravi Patel**

United States of America

**Scott Patten**

Canada

**Josef Patzak**

Czech Republic

**Vibhu Paudyal**

United Kingdom

**Anneleen Pauly**

Belgium

**Katherine Pawelczak**

United States of America

**Lucia Pawloski**

United States of America

**Raheem Paxton**

United States of America

**Brad Pearce**

United States of America

**Talima Pearson**

United States of America

**Michelle Peckham**

United Kingdom

**Nasheeta Peer**

South Africa

**Artemios Pehlivanidis**

Greece

**Manuel Peitsch**

Switzerland

**Tatjana Pekmezovic**

Serbia

**Rinaldo Pellicano**

Italy

**Karl Peltzer**

Thailand

**Lingtao Peng**

United States of America

**David Pennisi**

Australia

**Mary Louise Penrith**

South Africa

**Nilanka Perera**

Sri Lanka

**Rocio Perez-Portela**

Spain

**Stefania Perrucci**

Italy

**Keith Perry**

United Kingdom

**Michael Pfaller**

United States of America

**Penelope Phillips-Howard**

United Kingdom

**Armin Piehler**

Norway

**Robert Pilarski**

United States of America

**Paul Pilch**

United States of America

**Stefano Pileri**

Italy

**Andrea Pitzschke**

Austria

**Audrey Player**

United States of America

**Janice Pluth**

United States of America

**Nancy Podevin**

Germany

**Laura Jean Podewils**

United States of America

**David Poetker**

United States of America

**Supriya Pokkali**

United States of America

**Chiara Poletto**

France

**Mattia Poletto**

United Kingdom

**Gregor Pollach**

Malawi

**Palmiro Poltronieri**

Italy

**Carolina Pometti**

Argentina

**Jeanine Pommier**

France

**Ryan Potts**

United States of America

**Vanusa Pousada da Hora**

Brazil

**Ignasi Poves**

Spain

**Marleen Praet**

United States of America

**Vincent Prasanna**

India

**Anne-Catherine Prats**

France

**Lakdasa Premawardhana**

United Kingdom

**Andreas Prengel**

Germany

**Christopher Preston**

Australia

**David Proud**

Australia

**Paolo Provero**

Italy

**Mirja Puolakkainen**

Finland

**Enrico Purisima**

Canada

**Vilda Purutcuoglu**

Turkey

**Reed Pyeritz**

United States of America

**Man Qin**

China

**Hude Quan**

Canada

**Salman Qureshi**

Germany

**Nalini Radhakishun**

Netherlands

**Jasim Radhi**

Canada

**Petros Rafailidis**

Greece

**Georgia Ragia**

Greece

**Vafa Rahimi-Movaghar**

Iran

**Cláudia Rainho**

Brazil

**Sari Räisänen**

Finland

**Senaka Rajapakse**

Sri Lanka

**Kanniah Rajasekaran**

United States of America

**Mirjana Rajilic-Stojanovic**

Serbia

**Jasna Rakonjac**

New Zealand

**Manickam Ramalingam**

India

**Jose Manuel Ramos**

Spain

**Adriana Ramos Pereira**

United Kingdom

**Joana Ramos-Jorge**

Brazil

**Leonhard Ramseier**

Switzerland

**Maria Luigia Randi**

Italy

**Swati Rane**

United States of America

**Vibeke Rasch**

Denmark

**Shariq Rashid**

India

**Svein Rasmussen**

Norway

**Gurdeep Rastogi**

India

**Debolina Ray**

United States of America

**Josette Raymond**

France

**Abolfazl Razi**

United States of America

**Emma Redding**

United Kingdom

**Umesh Reddy**

United States of America

**Yogesh Reddy**

United States of America

**Bjorn Redfors**

Sweden

**Maggie Redshaw**

United Kingdom

**Alexis Regent**

France

**Marvin Reid**

Jamaica

**Patricia Reis**

Brazil

**Tercia Reis De Souza**

Mexico

**Mary Relling**

United States of America

**Jordi Rello**

Spain

**Louis Renault**

France

**James Renner**

Nigeria

**Zhuo Renying**

China

**Anastasia Renzi**

Italy

**Pedro Ribeiro**

Portugal

**Antonia Ribes**

Spain

**Debra Rickwood**

Australia

**Iman Ridda**

Australia

**Irmgard Riedmaier**

Germany

**Una Riekstina**

Latvia

**Gianluca Rigatelli**

Italy

**Matthew Riley**

United States of America

**Bertus Rima**

United Kingdom

**Nirmal Rimal**

Nepal

**Marit By Rise**

Norway

**Richard Rison**

United States of America

**Davide Risso**

United States of America

**Syed Riyaz-Ul-Hassan**

India

**Jane Robbins**

United States of America

**Annie Robert**

Belgium

**Christine Roberts**

Australia

**Peter Roberts**

United Kingdom

**Stephen Roberts**

United Kingdom

**Hugh Robertson**

United States of America

**Bernardino Roca**

Spain

**Maria Rocca**

Italy

**Jonas Rodrigues**

Brazil

**Manuel Rodriguez**

Argentina

**Sergio Alejandro Rodríguez-Quiroga**

Argentina

**David Rodríguez-Ruiz**

Spain

**Kristien Roelens**

Belgium

**Hendrik Roest**

Netherlands

**Scott Rogers**

United States of America

**Anabel Rojas**

Spain

**Carlos Rojas-Fernandez**

Canada

**Violeta Roka**

Greece

**Paolo Romano**

Italy

**Martha Cecilia Rosales Hernandez**

Mexico

**Hamilton Roschel**

Brazil

**Danielle Rose**

United States of America

**Richard Rosenquist**

Sweden

**Montse Roset**

Spain

**Adriano Rossi**

United Kingdom

**Stefan Rothenburg**

United States of America

**Pierre-Raphael Rothschild**

France

**Subarna Roy**

India

**Domenico Rubello**

Italy

**Kai Ruffmann**

Germany

**Enrique Ruiz De Gopegui**

Spain

**Susan Rumisha**

Tanzania

**Kim Russell**

United Kingdom

**Ligia Rusu**

Romania

**Sarah Rutstein**

United States of America

**Shomei Ryozawa**

Japan

**Osman Sabuncuoglu**

Turkey

**Sergio Sacca**

Italy

**Maike Sachs**

United States of America

**Brad Sagarin**

United States of America

**Sejal Saglani**

United Kingdom

**Ashis Saha**

India

**Tayfun Sahin**

Turkey

**Martina Sahre**

United States of America

**Rintaro Saito**

Japan

**Koji Sakai**

Japan

**Lesley Ann Saketkoo**

United States of America

**Adzura Salam**

Malaysia

**Cristiano Salata**

Italy

**Oyetunde Salawu**

Nigeria

**Gustavo Adolfo Salazar**

South Africa

**Sarah Saleem**

Pakistan

**Nikolaos Salemis**

Greece

**Cassandra Salgado**

United States of America

**Bharathi Salimath**

India

**Michael Samarkos**

Greece

**Arifi Samia**

Morocco

**Janneke N. Samsom**

Netherlands

**Alvaro Sanabria**

Colombia

**Andrew Sandford**

Canada

**Bjorn Sandstrom**

Sweden

**Sarah Sangster**

Canada

**Sathish Sankar**

India

**Walter Sanseverino**

Spain

**Sreevidya Santha**

United States of America

**Cristina Santos**

Portugal

**Alfredo Santovito**

Italy

**Gaetano Santulli**

United States of America

**Robert Saper**

United States of America

**Loredana Sarmati**

Italy

**Stefania Sarno**

Italy

**Sarah Sasson**

Australia

**Latha Satish**

United States of America

**Akira Sato**

Japan

**Hiroaki Satoh**

Japan

**Elena Savoia**

United States of America

**Susan Sayers**

Australia

**Valeria Scala**

Italy

**Hendrik Simon Schaaf**

South Africa

**Rene Schalk**

Netherlands

**Frieder Schaumburg**

Germany

**Debora Scheffel**

Brazil

**Mirte Scherpenisse**

Netherlands

**Frank Schiedel**

Germany

**Thomas Schiex**

France

**Raphael Schiffmann**

United States of America

**Antonio Schindler**

Italy

**Birgit Schittek**

Germany

**Marcus Schmidt**

Germany

**Robert Schmidt**

United States of America

**Michaela Schmidtke**

Germany

**Guido Schmiemann**

Germany

**Vicki Schnadig**

United States of America

**Caroline Schneeberger**

Netherlands

**Christa Schorr**

United States of America

**Matthias Schreiber**

Germany

**Ward Schrooten**

Belgium

**Gillian Scott**

Australia

**Stephen Seah**

Canada

**Anna Seale**

United Kingdom

**James Seddon**

United Kingdom

**Christopher Seebregts**

South Africa

**Teresa Seefeldt**

United States of America

**Janet Seeley**

Uganda

**Maria Mercedes Segura**

Spain

**Andrada Seicean**

Romania

**Elisabeth Seigner**

Germany

**Govindan Sadasivam Selvam**

India

**Andrzej Semczuk**

Poland

**Jeremiah Seni**

Tanzania

**Abiola Senok**

Saudi Arabia

**Efraim Serafetinides**

Greece

**Graham Serjeant**

Jamaica

**Junia Serra-Negra**

Brazil

**Siti Setiati**

Indonesia

**Ahmad Settin**

Saudi Arabia

**Teresa Sevilla**

Spain

**Guillermo Sguazza**

Argentina

**Kashif Shafique**

United Kingdom

**Shinil Shah**

United States of America

**Fahim Shaltout**

Egypt

**Seyed Morteza Shamshirgaran**

Australia

**Hariharan Shankar**

United States of America

**P Ravi Shankar**

Nepal

**Pratap Sharan**

India

**Aman Sharma**

India

**Lisa Sharwood**

Australia

**John Shaw**

New Zealand

**Yao Shen**

United States of America

**Sheela Shenoi**

United States of America

**Rezvi Sheriff**

Sri Lanka

**David Sherlock**

United Kingdom

**Yun-Bo Shi**

United States of America

**Xingjie Shi**

China

**Weibin Shi**

United States of America

**Zumin Shi**

Australia

**Hiroyuki Shibata**

Japan

**Joseph Shiber**

United States of America

**Jun Shigemura**

Japan

**Kuniaki Shirao**

Japan

**Yehuda Shoenfeld**

Israel

**Shifra Shvarts**

Israel

**Karl Sieber**

United States of America

**Sabrina Giovanna Signorini**

Italy

**Michael Silbermann**

Israel

**Jonas Silveira**

Brazil

**Vasileios Simaioforidis**

Netherlands

**Serap Simavli**

Turkey

**Elisabeth Simoes**

Germany

**Florian Simon**

Germany

**Sunder Sims-Lucas**

United States of America

**Bostjan Simunic**

Slovenia

**Alan Sin**

Singapore

**Andrew Sindone**

Australia

**Steven Singer**

United States of America

**Debra Singh**

Uganda

**Dheer Singh**

India

**Ravi Shankar Singh**

United States of America

**Surjit Singh**

India

**Tarundeep Singh**

India

**Alok Krishna Sinha**

India

**Nikolaos Sipsas**

Greece

**Eduardo Siqueira**

United States of America

**Cristina Maria Sirrenberg-Cruciat**

Germany

**Tesfaye Sisay Tessema**

Ethiopia

**Susan Sisson**

United States of America

**Vinoth Sittaramane**

United States of America

**Parco Siu**

Hong Kong

**Petros Skapinakis**

Greece

**Sheila Skeaff**

New Zealand

**Steven Skiena**

United States of America

**Nevena Skroza**

Italy

**Keith Skubitz**

United States of America

**Nives Škunca**

Switzerland

**Abdel Slama**

France

**Howard Slater**

Australia

**Elwira Sliwinska**

Poland

**Michel Slotman**

United States of America

**Hugo Matthias Smeets**

Netherlands

**Nada Smigic**

Serbia

**Brennan Smith**

Canada

**Jeramiah Smith**

United States of America

**Rebecca Smith**

United States of America

**Valerie Smith**

Ireland

**David G Smithard**

United Kingdom

**Katherine Smollett**

United Kingdom

**Rebecca Smyth**

United Kingdom

**John Snowdon**

Australia

**Mario Soares**

Australia

**Rodrigo Soares**

Brazil

**Kirstine Kobberoe Soegaard**

Denmark

**Shoshanna Sofaer**

United States of America

**Howard Sofen**

United States of America

**Rafael Solana**

Spain

**Stian Solem**

Norway

**Vincent Soler**

France

**Margrete Solheim**

Norway

**Maria Giuliana Solinas**

Italy

**Kerry Sondgeroth**

United States of America

**Ke Song**

China

**Joon Jin Song**

United States of America

**Shuhui Song**

China

**Alexei Sorokin**

France

**Manuel Soto**

Spain

**Eser Yildirim Sozmen**

Turkey

**Michael Sperling**

Germany

**Thambipillai Sri Paran**

Ireland

**Vibhuti Srivastava**

United States of America

**Willy Ssengooba**

Uganda

**Christopher Staley**

United States of America

**Carol Stanciu**

Romania

**Henriette Stavri**

Romania

**Lindsay Stead**

United Kingdom

**Clifford Steer**

United States of America

**Aggelos Stefos**

Greece

**Nils Stein**

Germany

**Herbert Steinbeisser**

Germany

**Nili Steinberg**

Israel

**Martin Steinberg**

United States of America

**Melinda Steis**

United States of America

**Matthew Stevens**

Australia

**Michael Stewart**

United States of America

**Christine Stirling**

Australia

**Ellen Stobberingh**

Netherlands

**Juan Carlos Stockert**

Spain

**Barbara Stoecker**

United States of America

**Melinda Stolley**

United States of America

**Michael Stoto**

United States of America

**Terry Stratton**

United States of America

**Richard Street**

United States of America

**Emily Chia-Yu Su**

Taiwan

**Ines Subota**

Germany

**Saravanan Subramaniam**

India

**Yoshinori Sugiura**

Japan

**Takashi Sugiyama**

Japan

**Munawar Sultana**

Bangladesh

**Fengjie Sun**

United States of America

**Nathan Sundgren**

United States of America

**Wing-Kin Sung**

Singapore

**Francesco Sunseri**

Italy

**Salim Surani**

United States of America

**Johari Surin**

Malaysia

**Alan Sustic**

Croatia

**Keiichiro Susuki**

United States of America

**Melissa Suter**

United States of America

**Brad Sutherland**

United Kingdom

**Yoshihiro Suzuki**

Japan

**Alessandra Swarowsky**

Brazil

**Waleed Sweileh**

Palestinian Territory

**Harold Swerdlow**

United Kingdom

**Deeba Syed**

United States of America

**Gulam Syed**

United States of America

**Bryan Sykes**

United States of America

**Maria Syrrou**

Greece

**Karol Szafranski**

Germany

**Gyorgy Szittya**

Hungary

**George Szmukler**

United Kingdom

**Sharon Sznitman**

Israel

**Demian Szyld**

United States of America

**Mozhgan Tabatabaei**

Iran

**Shams Tabrez**

Saudi Arabia

**Noriko Tada**

Japan

**Mioko Taguchi**

Japan

**Adel Taha**

Egypt

**Eman Taher**

Egypt

**Naoki Takahashi**

Japan

**Daisuke Takamatsu**

Japan

**Tohru Takata**

Japan

**Eiji Takeda**

Japan

**Masato Takeuchi**

Japan

**Takeshi Takeuchi**

Japan

**Marco Takita**

Brazil

**Ichiro Takumi**

Japan

**Hiroyuki Takuwa**

Japan

**Rakhshandra Talpur**

United States of America

**Usman Sumo Friend Tambunan**

Indonesia

**Tian Chye Tan**

Malaysia

**Haixu Tang**

United States of America

**Lili Tao**

China

**Mark Tarnopolsky**

Canada

**Glen Tattersal**

Canada

**Belaynew Taye**

Ethiopia

**David Taylor**

United Kingdom

**Stephanie Taylor**

United States of America

**Nicolas Tchitchek**

United States of America

**Anne Tebo**

United States of America

**Helder Tedeschi**

Brazil

**Anteneh Tesfaye Tefera**

Ethiopia

**Bin S. Teh**

United States of America

**Andrea Teixeira**

Brazil

**Scott Telfer**

United Kingdom

**Claire Telford**

United States of America

**Raymond Tellier**

Canada

**Tenzin Tenzin**

Bhutan

**Yasuo Terauchi**

Japan

**Gerasimos Terzis**

Greece

**Sergei Tevosian**

United States of America

**Seenivasagan Thangaraj**

India

**Florian Then Bergh**

Germany

**Georgios Theodoropoulos**

Greece

**Vasanthi Thevanesam**

Sri Lanka

**Ravi Thiagarajan**

United States of America

**Philip Thomas**

India

**Charlotte Thomas-Hawkins**

United States of America

**Alistair Thomson**

United Kingdom

**Kwai-Lin Thong**

Malaysia

**Lukar Thornton**

Australia

**Kamala Thriemer**

Australia

**Samuel Thumbi**

Kenya

**Bharat Thyagarajan**

United States of America

**X Cindy Tian**

United States of America

**Ellen Tijsse-Klasen**

Netherlands

**Patricia Tille**

United States of America

**Jozsef Timar**

Hungary

**Daisuke Tokuhara**

Japan

**Sue Tolin**

United States of America

**Hanna Tolonen**

Finland

**Iole Tomassini Barbarossa**

Italy

**Louis Tong**

Singapore

**Kwasi Torpey**

Nigeria

**Javier Torres**

Mexico

**Solveig Tosi**

Italy

**Bruno Tota**

Italy

**Abdelhalim Trabelsi**

Tunisia

**Thach Tran**

Vietnam

**Jason Treberg**

Canada

**Carla Treloar**

Australia

**Omar Triana**

Colombia

**Konstantinos Triantafyllou**

Greece

**Nikos Tsagias**

Greece

**Anastasia Tsagkarakou**

Greece

**Chieh-Chih Tsai**

Taiwan

**Yao Hung Tsai**

Taiwan

**Stephen Tsang**

United States of America

**Iraklis Tsangaris**

Greece

**Rea Tschopp**

Switzerland

**Alexandra Tsigginou**

Greece

**Sotirios Tsiodras**

Greece

**Jun Tsugawa**

Japan

**Vedat Turhan**

Turkey

**Kamuran Türker**

Turkey

**Paul Turner**

Cambodia

**Jaroslaw Tyburski**

Poland

**Stavros Tyritzis**

Greece

**Flora Tzelepis**

Australia

**Ekong Udoh**

Nigeria

**Yusaku Uga**

Japan

**Gabriele Uhl**

Germany

**Kingsley Nnanna Ukwaja**

Nigeria

**Glen Ulett**

Australia

**Nawaraj Upadhaya**

Nepal

**Stephan Urban**

Germany

**Constantine Vagianos**

Greece

**Philippe Vago**

France

**Edi Vaisbuch**

United States of America

**Michael Todd Valerius**

United States of America

**Natalia Vallianou**

Greece

**Olga van den Akker**

United Kingdom

**Dimitri Van der Linden**

Netherlands

**Nathalie van der Mee-Marquet**

France

**Hanneke van der Meide**

Netherlands

**Andre Van Der Merwe**

South Africa

**Eveline van der Veer**

Netherlands

**TM van Ginhoven**

Netherlands

**Joost J. Van Middendorp**

Netherlands

**Gijs Van Pottelbergh**

Belgium

**Marleen Van Troys**

Belgium

**Nico Van Zandwijk**

Australia

**Timothy VanWagoner**

United States of America

**Vijayavlakshmi Varma**

United States of America

**Marta Vascellari**

Italy

**Dimitra Argyro Vassiliadi**

Greece

**Ryash Vather**

New Zealand

**Gilberto Vaughan**

United States of America

**Rukumani Devi Velayuthan**

Malaysia

**Sander Veldhuyzen Van Zanten**

Canada

**Martina Vendrame**

United States of America

**Danae Venieri**

Greece

**Kavita Venkataraman**

Singapore

**Francisco J Vera-Garcia**

Spain

**Marina Vercelli**

Italy

**Gilles Vergnaud**

France

**Renate Verkaik**

Netherlands

**Praveen Verma**

India

**Michael Verzi**

United States of America

**Lene Karine Vestby**

Norway

**Flavie Vial**

Switzerland

**Katara Vidyalakshmi**

India

**Carla Vignaroli**

Italy

**Joseph Vinetz**

United States of America

**Vincenzo Viscosi**

Italy

**Leo Visser**

Netherlands

**Gonzalo Vitagliano**

Argentina

**Petros Vlastarakos**

United Kingdom

**Christian Voigt**

Germany

**Yskert von Kodolitsch**

Germany

**Frauke von Versen-Höynck**

Germany

**Maarten Vonhof**

United States of America

**Jean Paul Vonsattel**

United States of America

**Stefan Vordenbäumen**

Germany

**Juergen Vormann**

Germany

**Anna Vossbeck-Elsebusch**

Germany

**Varaporn Vuddhakul**

Thailand

**Bojan Vujkovac**

Slovenia

**Elizabeth Wager**

United Kingdom

**Roland Wagner**

Germany

**Kenneth Wagner**

United States of America

**Thomas Wakefield**

United States of America

**Matthew Wakefield**

Australia

**Denis Walsh**

United Kingdom

**Xiang Wan**

Hong Kong

**Ying Wan**

United States of America

**Qiuzhen Wang**

China

**Dafu Wang**

United States of America

**Debin Wang**

China

**Jing Wang**

United States of America

**Gangshi Wang**

China

**Gaiqing Wang**

China

**Yuhong Wang**

United States of America

**Wei Zhi Wang**

United Kingdom

**WenFang Wang**

United States of America

**Xin Wang**

United States of America

**Xuefeng Wang**

United States of America

**Robert YL Wang**

Taiwan

**Judson Ward**

United States of America

**Sarah Ward**

Australia

**Clemens Warnke**

Germany

**Knut Waterloo**

Norway

**Claus Wedekind**

Switzerland

**Carl Weems**

United States of America

**Kosala Gayan Weerakoon**

Sri Lanka

**David Weetman**

United Kingdom

**Hua Wei**

United States of America

**Ralf Weigel**

United Kingdom

**Shannon Weigum**

United States of America

**Chang Wei-Kuo Chang**

Taiwan

**Lauren Weinstock**

United States of America

**Jan Weis**

Sweden

**Laura Welch**

United States of America

**Michel Wensing**

Netherlands

**Andrea Wessell**

United States of America

**Johanna Westra**

Netherlands

**John Wheeler**

United States of America

**David Whitford**

Bahrain

**John Whyte**

United States of America

**Suwit Wibulpolprasert**

Thailand

**Laetitia Wilkins**

Switzerland

**Merlin Willcox**

United Kingdom

**Michele Williams**

United States of America

**Sandra Williams-Phillips**

Jamaica

**Elizabeth Wilson**

New Zealand

**Tomasz Wojdacz**

Sweden

**Randall Wolcott**

United States of America

**Philip Wong**

Canada

**Kum Thong Wong**

Malaysia

**Wing-Cheong Wong**

Singapore

**Charles Wood**

United States of America

**Jiunn-Jong Wu**

Taiwan

**Ning Wu**

United States of America

**Jia-Feng Wu**

Taiwan

**Jie Wu**

United States of America

**Kevan Wylie**

United Kingdom

**Alidianne Xavier**

Brazil

**Zheng Xia**

United States of America

**Ying Xian**

United States of America

**Dingli Xu**

China

**Shu Xu**

United States of America

**Xianzhong Xu**

United States of America

**Han Xue**

China

**Yusuf Yagmur**

Turkey

**Yusuf Yakupogullari**

Turkey

**Emre Yalcinkaya**

Turkey

**Wing Cheong Yam**

China

**Toshihiko Yamada**

Japan

**Saburo Yamamoto**

Japan

**Aparecida Yulie Yamamoto**

Brazil

**Ching-Huei Yang**

Taiwan

**Tianbing Yang**

United States of America

**Zhaogang Yang**

United States of America

**Nan-Ping Yang**

Taiwan

**Yoshihiko Yano**

Japan

**Maria Yazdanbakhsh**

Netherlands

**Zhiwei Ye**

United States of America

**Maurice Yé**

Burkina Faso

**Chen-Hsiang Yeang**

Taiwan

**Gursel Yilmaz**

Turkey

**Ozlem Yilmaz**

Turkey

**Zerrin Yilmaz Celik**

Turkey

**Eppie Yiu**

Australia

**Gregory Yochum**

United States of America

**Simon Yolung**

Canada

**Yasushi Yoshino**

Japan

**Erfan Younesi**

Germany

**Pampee Young**

United States of America

**Muhammad Yousafzai**

Pakistan

**Chang-En Yu**

United States of America

**David Yu**

United States of America

**Hsiang-Hsuan Yu**

United States of America

**Zhu Yuanjie**

China

**Yasim Yusof**

Malaysia

**Arseniy Yuzhalin**

Russian Federation

**Aziz Zaanan**

France

**Iñigo Zabalgogeazcoa**

Spain

**Balaji Zacharia**

India

**Armin Zadeh**

United States of America

**Olaf Zagólski**

Poland

**Khalequ Zaman**

Bangladesh

**Michal Zamecnik**

Czech Republic

**Vicente Zanon-Moreno**

Spain

**Shelley Zansky**

United States of America

**Apostolos Zaravinos**

Sweden

**Tao Zeng**

China

**Xiaowei Zhan**

United States of America

**Rui Zhang**

United States of America

**Shicui Zhang**

China

**Yaobi Zhang**

Senegal

**Pengjun Zhang**

China

**Yongliang Zhang**

United States of America

**Zhancheng Zhang**

United States of America

**Min Zhao**

United States of America

**Cheng Zheng**

China

**Guoping Zheng**

Australia

**Cuncong Zhong**

United States of America

**Demin Zhou**

China

**Hong-Zhang Zhou**

China

**Chao-Dong Zhu**

China

**Qihui Zhu**

United States of America

**Yimin Zhu**

China

**Ralph Ziegler**

Germany

**Eleni Zografos**

Greece

**Rita Zrenner**

Germany

**Ilaria Zucchiatti**

Italy

